# Optimization of breeding program design through stochastic simulation with kernel regression

**DOI:** 10.1093/g3journal/jkad217

**Published:** 2023-09-23

**Authors:** Azadeh Hassanpour, Johannes Geibel, Henner Simianer, Torsten Pook

**Affiliations:** Department of Animal Sciences, Center for Integrated Breeding Research, Animal Breeding and Genetics Group, University of Goettingen, 37075 Goettingen, Germany; Department of Animal Sciences, Center for Integrated Breeding Research, Animal Breeding and Genetics Group, University of Goettingen, 37075 Goettingen, Germany; Institute of Farm Animal Genetics, Friedrich-Loeffler-Institut, 31535 Neustadt, Germany; Department of Animal Sciences, Center for Integrated Breeding Research, Animal Breeding and Genetics Group, University of Goettingen, 37075 Goettingen, Germany; Department of Animal Sciences, Center for Integrated Breeding Research, Animal Breeding and Genetics Group, University of Goettingen, 37075 Goettingen, Germany; Wageningen University & Research, Animal Breeding and Genomics, 6700 AH Wageningen, Netherlands

**Keywords:** optimization, resource allocation, kernel regression, genetic gain, inbreeding

## Abstract

In recent years, breeding programs have increased significantly in size and complexity, with various highly interdependent parameters and many contrasting breeding goals. As a result, resource allocation in these programs has become more complex, and deriving an optimal breeding strategy has become increasingly challenging. To address this, a common practice is to reduce the optimization problem to a set of scenarios that differ only in a few parameters and can therefore be analyzed in detail. The goal of this article is to provide a framework for the numerical optimization of breeding programs that goes beyond the simple comparison of scenarios. For this, we first determine the space of potential breeding programs only limited by basic constraints like the budget and housing capacities. Subsequently, the goal is to identify the optimal breeding program by finding the parametrization that maximizes the target function by combining different breeding goals. To assess the value of the target function for a parametrization, we propose using stochastic simulations and the subsequent use of a kernel regression method to cope with the stochasticity of simulation outcomes. This procedure is performed iteratively to narrow down the most promising areas of the search space and perform more and more simulations in these areas of interest. In a simplified example applied to a dairy cattle program, our proposed framework has shown its ability to identify an optimal breeding strategy that aligns with a target function aiming at genetic gain and genetic diversity conservation limited by budget constraints.

## Introduction

Designing a breeding program is a complex task that requires considering multiple interdependent breeding objectives (Berry 2015; Hickey *et al*. 2017). Limited resources, both financial and practical, impose restrictions on the scale and scope of breeding activities (Henryon *et al*. 2014). As a result, breeders face the challenge of making various decisions to maximize resource utilization by prioritizing specific breeding objectives and optimizing strategies to achieve the best possible outcome within the given constraints.

Over the years, various methods using quantitative genetic theory have been developed to estimate the impact of specific changes on the breeding program. For example, the breeder’s equation (Lush 1947; Falconer and Mackay 1996) provides an estimate of how selection intensity, generation interval, and prediction accuracy will impact the response to selection. Based on this, several tools like ZPLAN (Täubert *et al*. 2010), SelAction (Rutten *et al*. 2002), and MTINDEX by J. van der Werf (see http://www.personal.une.edu.au/˜jvanderw/software.htm) have been developed to predict multiple breeding decisions with each other and estimate the expected response to selection, enabling strategic resource allocation, and making informed decisions.

While this formula provides valuable insights for optimizing breeding programs, its applicability is often restricted to simplified scenarios, and its limited generalizability hinders its application to modern programs that involve numerous interdependent parameters. In such programs, comparing diverse breeding schemes with different objectives becomes challenging. Even minor adjustments in one parameter can significantly affect multiple aspects of the program (Henryon *et al*. 2014; Simianer *et al*. 2021).

With increasing computational power, stochastic simulations have emerged as a valuable enhancement for analyzing breeding programs with various software solutions available (Sargolzaei and Schenkel 2009; Faux *et al*. 2016; Liu *et al*. 2018; Pook *et al*. 2020). This provides additional challenges but also opportunities. On the one hand, it is not possible to directly derive the expected outcome/value of key metrics of the breeding scheme (e.g. as done with the breeder’s equation). On the other hand, there are many benefits of using stochastic simulations, where it is often preferred over a deterministic simulation approach. To name a few: (1) their ability to incorporate multistage selection and rates of inbreeding more easily (de Roos *et al*. 2011), (2) simulation of populations over a specific time considering variability and stochastic events that can influence the population dynamics (de Roos *et al*. 2011; Lillehammer *et al*. 2011; Mc Hugh *et al*. 2011), and (3) instead of considering cohorts of individuals like in the traditional gene-flow model (Hill 1974), stochastic simulations simulate individuals and all breeding actions, which also allows for changes in the accuracy of breeding values by considering SNP effects and the genetic distance between the reference population and the predicted population (Lillehammer *et al*. 2011; Mc Hugh *et al*. 2011), allowing for more flexibility and in-depth modeling of modern breeding schemes. Nevertheless, as the expected outcome of a breeding scheme cannot be calculated deterministically, optimizing breeding programs using stochastic simulation has to cope with the additional challenge of stochasticity (Amaran *et al*. 2016).

Due to the advantages mentioned above, stochastic simulation has been utilized in different studies to execute such simulations for specific breeding actions and evaluate various breeding strategies. Several breeding programs aim to maximize genetic gain while minimizing the rate of inbreeding, which can have negative consequences on genetic diversity and overall program success. To achieve this balance, various methods and algorithms have been developed for different optimization strategies. Depending on the study, the focus of optimization can range from selection and mating decisions (Moeinizade *et al*. 2019, 2022; Kinghorn *et al*. 2022), weighting between different breeding objectives/traits (Gorjanc and Hickey 2018; Duenk *et al*. 2021), the use of optimal contribution selection (Woolliams *et al*. 2015; Wellmann 2019), or maintenance of genetic diversity (Pryce and Daetwyler 2012).

Unlike deterministic calculations, the output of a stochastic simulation of breeding programs is the realization of a stochastic process. As a result, optimization cannot be achieved by straightforwardly maximizing a formula through derivative calculations. Therefore, applying mathematical optimization techniques, such as gradient descent (Kiefer and Wolfowitz 1952), simulated annealing (Kirkpatrick *et al*. 1983), genetic algorithms (Holland 1992), or Bayesian optimization (Schonlau 1998), becomes increasingly difficult. This is primarily due to the resource-intensive nature of stochastic simulations, as evaluating the optimization target for a single parametrization can require substantial computing time compared to the optimization algorithm itself. Due to the complex system architecture and the various sources of uncertainty, combined with the high number of parameters to consider, it becomes computationally challenging to simulate each potential breeding scheme and directly derive the optimal one. Therefore, to overcome these challenges, performance evaluation of the breeding program and designing breeding plans using decision frameworks are usually limited to a couple of potentially interesting scenarios, which are then simulated and compared against each other (Wensch-Dorendorf *et al*. 2011; Esfandyari *et al*. 2015; Gaynor *et al*. 2017; Büttgen *et al*. 2020; Pook *et al*. 2021).

Another commonly used technique for optimization is the use of a grid search algorithm (Longin *et al*. 2006; Gordillo and Geiger 2008; Mi *et al*. 2014, 2016; Pook *et al*. 2021). However, while grid search offers an acceptable solution to the problem in many smaller applications, it becomes inefficient for a large number of parameters. This inefficiency arises due to the exponential increase in computational time required to define and evaluate a grid of possible parameter combinations. Consequently, this often leads to the use of a very sparse grid, limiting its effectiveness in such scenarios.

While several algorithms and techniques have been developed to address optimization problems and resource allocation in breeding programs, the effectiveness of many existing algorithms can vary depending on specific problem characteristics. Although these frameworks offer valuable insights into setting up a breeding program, the aim of breeding program optimization so far has been focused on custom-designed programs, with aspects chosen specifically for the analysis of the particular breeding program at hand.

The goal of this article is explicitly not the optimization of the design of a specific breeding program but to provide a general framework to formalize the structure of a breeding program into a general optimization problem that in turn can be optimized. The particular focus here is on providing a framework to handle the randomness in stochastic simulations for the optimization of breeding program design, which makes it challenging for conventional optimization solvers to converge to optimal solutions.

## Materials and methods

### General pipeline for optimizing breeding programs

**Fig. 1.**
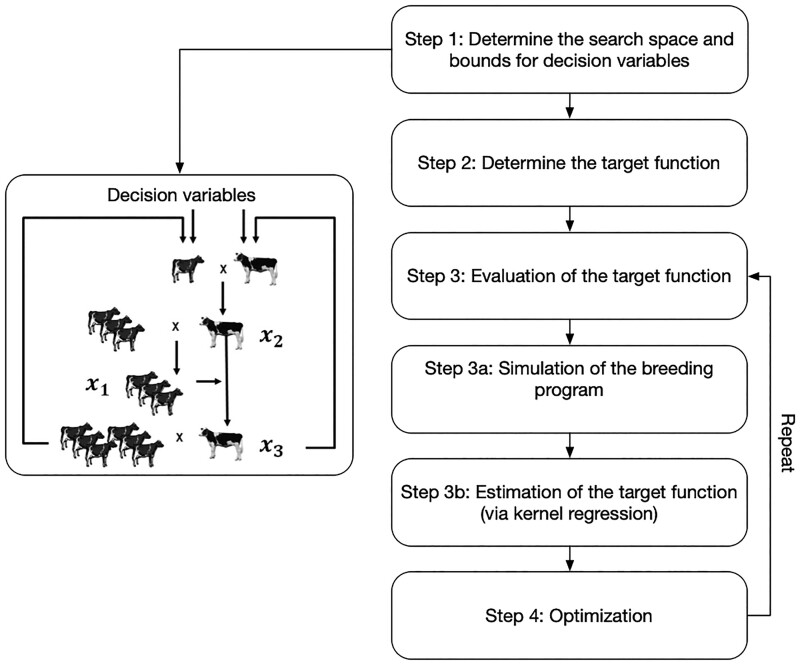
Procedure proposed for optimization via simulation for a breeding strategy.

We will propose a general pipeline for optimizing breeding programs in the following. A schematic overview of the different steps is given in Fig. 1. Subsequently, we will discuss the individual steps of the pipeline in more detail and discuss them along a classical dairy cattle scheme with a detailed description given in Supplementary File S1 and a summary of the simulation process outlined in Supplementary Table S1. Note that the breeding scheme described here is an oversimplified representation of reality and should only be seen as a toy example. However, the principles outlined can be adapted and applied to even more complex breeding schemes that involve multiple breeding steps, advanced selection techniques, varying cost structures, and other relevant factors.

#### Step 1: Determine the search space and bounds for decision variables

The first step in an optimization process is to identify which parameters can be changed and are subject to optimization and what range of values these can take. In the case of the dairy cattle example, we want to consider three variables $x=(x_{1},x_{2},x_{3})$ for optimization with $x_{1}$ being the number of test daughters, $x_{2}$ being the number of test bulls and $x_{3}$ as the number of selected sires, and these numbers must be non-negative integers. For a breeding program, the associated costs will most commonly provide basic boundaries to the considered variable. For our analysis, we will disregard any potential effects of economies of scale and instead assume a fixed annual budget of 10M €. Additionally, we will consider a housing cost of 3,000 € per bull and 4,000 € per cow.

As the entire search space will be considered for optimization, it can additionally make sense to limit parameters to a range of reasonable values based on prior expectations. For our analysis, we will focus on breeding schemes where the number of test bulls ranges from 100 to 700, and the number of selected sires ranges from 3 to 30. We chose to limit our consideration to this range as other designs are expected to be less efficient, and we excluded them to save computing time. If the optimal solution is found in a corner solution, these constraints may need to be adjusted and softened to ensure that the best solution will not be missed. Similarly, one could imagine practical limitations like a maximum housing capacity in the stable


$$\begin{array}{c} x_{1}+x_{2}+x_{3}\geq 0\\ 100\leq x_{2}\leq 700\\ 3\leq x_{3}\leq 30\;\\ 4,000x_{1}+3,000x_{2}-10,000,000\leq 0 \end{array}$$

#### Step 2: Determine the target function

To perform any type of optimization it is required to have a well-defined target function, hence a breeding goal with the final objective being to find the parameterization in our search space to maximize this target. Options for this are basically endless, and from our experience, even breeding companies typically struggle to describe what their overall and concrete breeding goal is. Consequently, this can range from a purely economic description of what financial impact a given breeding program has to minimize money spent to fulfill a set amount of genetic gain or to maximize genetic gains in a certain time frame plainly. This requires analyzing the output of the stochastic simulations, which in contrast to the simulations themselves, should be computationally relatively cheap and thus allow for limitless complexity in the target function.

In our toy example, we want to obtain high genetic gain while also maintaining genetic diversity in the population. For this, we are considering the following target function *m*:


$$m(x_{1},x_{2},x_{3})=g(x_{1},x_{2},x_{3})-50\times f(x_{1},x_{2},x_{3})$$

with *g* being the resulting genetic gain and *f* being the inbreeding level in newly born animals after 15 years of breeding. *g* is calculated based on the true underlying genomic values of individuals [get.bv() in MoBPS (Pook *et al*. 2022)], and *f* is calculated based on kinship [kinship.emp.fast() in MoBPS (Pook *et al*. 2022)].

In this example, we chose a scaling factor of 50 for *f* to give approximately equal importance to both breeding goals. It is important to note that this choice was arbitrary and based on the range of values observed in *f* and *g*. The appropriate choice of the weighting factor will vary depending on the specific breeding scheme and goals. In the end, in our example, this allows the two objective functions to be compared and allows for a more balanced consideration of their trade-offs. To account for various time horizons in the breeding scheme, an alternative approach is to incorporate not only genetic gains after 15 years but also consider a composite target function that includes genetic gains after 1, 2, 3, 5, and 10 years.

#### Step 3: Evaluation of the target function

In contrast to most other fields, evaluating the target function itself is the main computational bottleneck of the optimization procedure. We split this up into first simulating the breeding program, corresponding to sampling a realization of the target function for a given parametrization, and estimating/approximating the target function based on these outputs.

#### Step 3a: Simulation of the breeding program

The process of simulating a breeding program is often overlooked in many scientific manuscripts focusing on simulation studies, but setting up this script can be seen as one of the key components of any simulation study. For a general optimization, it is required to write the simulation script in a flexible and general way to handle all parameter settings within the considered search space. For instance, when exploring the use of optimum genetic contribution (OGC) to preserve genetic diversity within a breeding program (Quinton and Smith 1995), the simulation script must be designed to adapt based on the chosen parameterization. It should be capable of incorporating OGC when applicable and excluding it when irrelevant. Additionally, if one is investigating the utilization of admixture arising from a founding population comprising various subpopulations, the simulation script should be developed to include the coexistence of all subpopulations throughout the simulation process (Corbett-Detig and Jones 2016). Furthermore, if certain scenarios involve generating specific cohorts, the code should be flexible enough to handle cases where these cohorts may or may not be present. One way to achieve this flexibility is by utilizing binary parameters that control whether a particular breeding action is executed or skipped within the simulation script.

In our example, we utilized the R package MoBPS (Pook *et al*. 2020) to perform the stochastic simulation of the breeding scheme. This package offers a flexible environment and a wide range of preimplemented functions that facilitate the implementation of the intended breeding action. Given the simplicity of our toy example, the implementation process was fairly straightforward (Supplementary File S3). However, for more complex breeding programs, the general procedure remains the same, albeit with longer computing times and additional considerations when preparing the simulation script.

#### Step 3b: Estimation of the target function (via kernel regression)

If unlimited computing power was available, one could simulate each potential parameterization of the breeding program multiple times. These simulations would then be evaluated against the target, enabling the identification of the breeding scheme that maximizes the desired outcome. The outcomes of our simulation are just realizations of a stochastic process and not direct calculations of *g*, *f*, and *m*, respectively. Instead, we want to use these realizations to calculate estimators $\hat{g}$, $\hat{f}$, and $\hat{m}$.

Given the large number of potential settings and the computational demands associated with simulations, achieving this level of exhaustive evaluation is practically unfeasible. Additionally, due to the high level of stochasticity in the results, the target function does not behave well when employing relatively simplistic means of approximation, such as linear interpolation, and this hinders the application of downstream optimization techniques [e.g. utilizing gradient descent as an optimization technique necessitates a well-defined derivative and may encounter difficulties such as local maxima arising from stochastic outliers (Shah *et al*. 2020)].

We here propose the following approximation pipeline for *m*. To initialize this procedure, it is necessary first to simulate potential parametrizations of the breeding scheme with broad coverage of the search space. In our particular example, values for $x_{2}$ and $x_{3}$ were drawn from a uniform distribution, and $x_{1}$ was subsequently calculated based on the budget constraint. In our study, we sampled 60,000 potential parametrizations for the breeding program. This sample size was chosen considering the relatively low computing time of the toy example and the significant influence of stochasticity observed. It is important to note that the number of simulations conducted will depend on the available computing time and with more simulations a better initial approximation can be achieved.

For the approximation of $\hat{g}$, $\hat{f}$, and $\hat{m}$, we propose using kernel regression via a Nadaraya–Watson estimator (Nadaraya 1964; Watson 1964), which provides a locally weighted average with weighted derived based on the distance of each input parametrization to the target value ($x_{1}$, $x_{2}$, $x_{3}$) to estimate the underlying function. We can define the estimator of *m* as:


$$\begin{array}{rl} E(Y|x & =(x_{1},x_{2},x_{3}))=\hat{m}(x_{1},x_{2},x_{3})\\ & =\frac{\sum y_{i}\times K(\frac{x_{1}-x_{i,1}}{h_{1}},\frac{x_{2}-x_{i,2}}{h_{2}},\frac{x_{3}-x_{i,3}}{h_{3}})}{\sum K(\frac{x_{1}-x_{i,1}}{h_{1}},\frac{x_{2}-x_{i,2}}{h_{2}},\frac{x_{3}-x_{i,3}}{h_{3}})}. \end{array}$$

Note that *Y* is a random variable with an expected value $m(x_{1},x_{2},x_{3})$ and unknown variance. $y_{i}$ is the realization of stochastic simulations for our three input parameters $\left(x_{i,1},x_{i,2},x_{i,3}\right)$. For each value of *x*, the locally weighted average of the $y_{i}$ is computed with weights given by the used kernel function. We here use a multivariate Gaussian kernel with independent individual components, resulting in the following:


$$K(x_{1},x_{2},x_{3})=K_{1}(x_{1})K_{2}(x_{2})K_{3}(x_{3})$$

with


$$K_{i}(x)=\frac{1}{\sqrt{2\pi }}\exp\;\left(-\frac{x^{2}}{2}\right),$$

where *x* is the distance between the query and a data point in the input space, the kernel function is linked to the smoothing parameter bandwidth *h*, which controls the weight given to each simulation in the smoothing procedure. A smaller bandwidth means that the $x_{i}$ closer to *x* will have higher relative weights, and therefore their $y_{i}$ values will have more influence on the estimate.

For the choice of the initial bandwidth, one could consider using cross-validation (Hardle and Marron 1985; Jones *et al*. 1996) or estimation of the variance in the given parameter (Brockmann *et al*. 1993). For our toy example, we chose the initial bandwidth based on visual inspection to obtain a relatively well-based function without over-smoothing with $\left(h_{1}=30,h_{2}=30,h_{3}=1\right)$ for *g*. In order to mitigate biases in the estimation process caused by the higher weighting of scenarios with varying selection intensities during cases of high selection intensity, the bandwidth values for *f* were divided by 3. This adjustment was made specifically to address the major changes in the realization of *f* for the low number of selected sires $x_{3}$ (high selection intensity).

#### Step 4: Optimization

Results from the kernel regression provide an approximated target function that offers the advantages of quick evaluation for a given parametrization and reasonable behavior. This approximation enables the utilization of diverse optimization techniques. With a limited number of parameters, it will often just be possible to evaluate all parametrizations (as demonstrated in our toy example using the brute-force approach). However, one could also consider gradient descent [optimr by Nash (2019)], simulated annealing [GenSA by Xiang *et al*. (2013)], genetic algorithms [GA by Scrucca (2013, 2017)], differential evolution [DEoptim by Mullen *et al*. (2011)]. See Schwendinger and Borchers (2023) for an overview of methods for mathematical optimization.

Moreover, it is important to note that the target function is not directly observed but rather approximated. Therefore, it becomes crucial to utilize the results of the approximation in order to identify the most promising regions within the search space by running additional simulations in those areas. With the additional simulations, the approximation of the target function can be improved, leading to a reduction in estimation bias. This improvement can be achieved by decreasing the bandwidth of the kernel regression, which in turn, reduces bias. Additionally, conducting more simulations helps to decrease the variance of the estimation, contributing to a more accurate approximation overall. Hence, optimization is performed iteratively until a stable point is reached in the estimated maximum of those identified areas. At this stage, further exploration is unlikely to yield significant changes, indicating that convergence has been achieved.

### Comparison of approximation techniques

To assess and compare the performance of the suggested kernel regression method, we compared optimization results obtained through plain linear interpolation [interp by Akima *et al*. (2022)]. For evaluation purposes, we generated a dataset of 30,000 samples from the original dataset, which contained 60,000 simulations, using bootstrapping. This process was repeated 100 times to ensure reliability and consistency. We then compared the two metrics based on the average value of the target function, as estimated from the full set of simulations, and the average distance of the suggested optima from the same full set.

### Number of simulations

The inherent randomness in stochastic simulation can lead to significant variations between individual simulations. To determine the precision of our results within a single iteration, we evaluated the required number of simulations. For this purpose, we performed simulations using different sample sizes (1,000, 10,000, 20,000, 60,000). Subsequently, we compared the likelihood of the estimated optima occurring in the search space using varying numbers of simulations. Further details of this method can be found in Supplementary File S2.

### Computing time for simulation

A server cluster with Intel Platinum 9242 (2X48 core 2.3 GHz) processors was used for this study. Simulations were performed on the single core and required ∼19 min and 5GB RAM peak memory usage per simulation (MoBPS Version: 1.10.40).

## Results

### Optimization via visual inspection

**Fig. 2.**
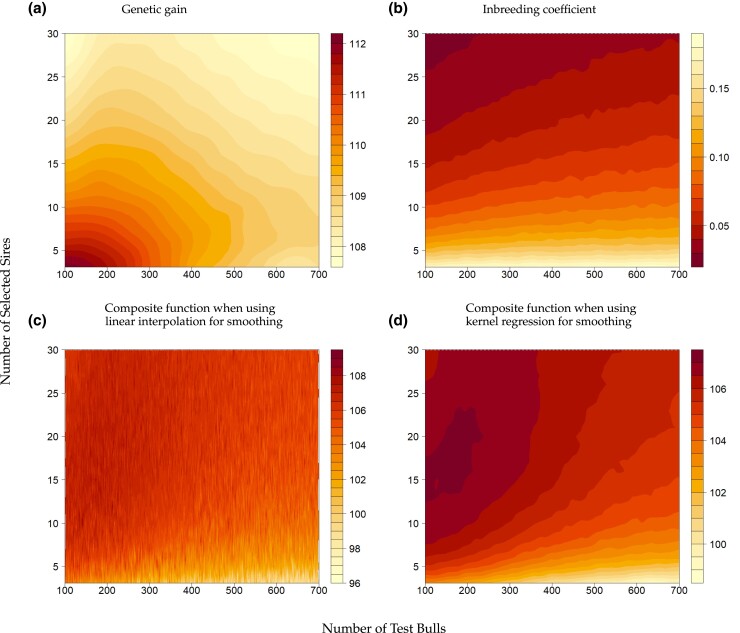
Visualization for Nadaraya–Watson estimator for a) genetic gain $\left(\hat{g}\right)$ with a bandwidth $\left(h_{2}=30,h_{3}=1\right)$, b) inbreeding coefficient $\left(\hat{f}\right)$ with a bandwidth of $\hat{g}/3$, c) composite function $\hat{m}$ based on 60,000 simulations when using linear interpolation for smoothing, d) composite function $\hat{m}$ based on 60,000 simulations when using kernel regression for smoothing. The colors represent the relative value of the target function, with dark red showing the favorable outcome of the target function.

In the following section, we will present the results obtained by applying our optimization pipeline to the toy example. Despite conducting as many as 60,000 simulations, the approximated target function derived from linear interpolation did not exhibit the desired behavior. Thus, this makes the application of the standard optimization techniques very challenging (Fig. 2c). By applying kernel regression to the target function, we significantly improved its smoothness, and we observed the highest value for the target function at (2377,163,18), yielding the optimal value of 107.1139 (Fig. 2d).

When looking into the individual components of the target function, we observe that genetic gain is maximized by using as many test daughters as possible to ensure high prediction accuracy and minimizing the number of used selected sires for a high selection intensity (2425, 100, 3) with a genetic gain of 12.2 (Fig. 2a). On the contrary, minimizing average kinship involves selecting as many selected sires as possible from a smaller pool of test bulls (2425, 100, 30), resulting in an inbreeding coefficient of 0.022 (Fig. 2b). Suppose those individual components would be the sole optimization goal, one should investigate to loosen the initially defined constraints. However, if these constraints are met in practice, one could stop the optimization process here and consider these corner solutions as the best optima.

**Fig. 3.**
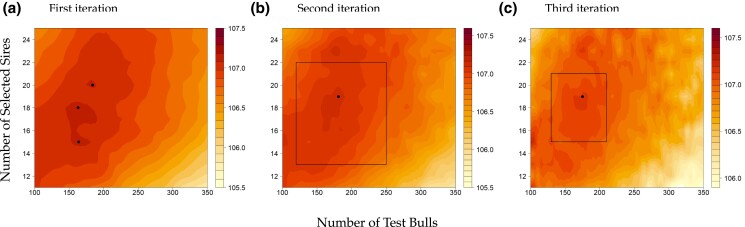
Smoothed surface contour map for the composite function of a) full space (zoom-in plot of Fig. 2d to show three local optima): First iteration with a bandwidth $\left(h_{2}=30,h_{3}=1\right)$, b) second iteration with a bandwidth $\left(h_{2}=15,h_{3}=0.5\right)$, c) third iteration with a bandwidth $\left(h_{2}=7.5,h_{3}=0.25\right)$. The indicated bandwidth here refers to the bandwidth for *g*, and this bandwidth for *f* in all iterations was divided by three. Dots indicate local maxima and the area inside of the squares indicates the search space in the current iteration. The area outside the square shows the prominent effect of the bandwidth and the variance of our estimates, which is related to the number of simulations used to estimate the kernel function. The colors represent the relative value of the target function, with dark red showing the favorable outcome of the target function.

However, when dealing with the composite function, the optimization process becomes less straightforward. It should be noted that we intentionally selected weights in the target function to ensure that the optima are within our search space. When zooming on the plot of the composite function, we can identify three local optima, where two different local maxima with similar values (2375, 166, 15) and (2362, 184, 20) lead to a value for the target function of 107.1073 and 107.0834, respectively. Thus, this first iteration is insufficient to narrow down the absolute maximum. However, it allows us to narrow down the search space for subsequent iterations to $120\leq x_{2}\leq 250$ and $13\leq x_{3}\leq 22$, as all three values fall within this area (Fig. 3a).

As a result, an additional 50,000 simulations were conducted in the second iteration, focusing only on the promising areas of the search space (areas inside of the black square in Fig. 3b). In the second iteration, the search space size was significantly reduced by narrowing the grid from a 27×600 grid to a 9×130 grid, resulting in a 14-fold decrease in the search space. As the number of simulations increased within the defined window size for calculating the kernel function, the variance in the estimation of the target function decreased compared to the first iteration. This allows us to reduce the bandwidth, allowing for a better approximation of our target function. Therefore, the bandwidth was halved, which resulted in the best solution 107.1205 from optimization, suggesting (2364, 181, 19) as the optimum.

In the third iteration, we conducted an additional 12,000 simulations by reducing the grid from 9×130 to 6×80, where the size of the search space was decreased by 2-fold. The reduction in the search space size enabled an increase in the number of observations within the defined window and a reduced bandwidth by half. The optimization suggests (2368, 175, 19) indicating stabilization of the optima with the best value of 107.1399 (Fig. 3c). Note that as no further simulations were run outside of the areas of interest, the chosen bandwidth will be too small to reliability estimate *m* outside the new search space as seen in Fig. 3c. If a specific region shows high potential, it may be worthwhile to run additional simulations in those previously disregarded regions. This will be particularly relevant for more complex optimization problems with more parameters.

**Fig. 4.**
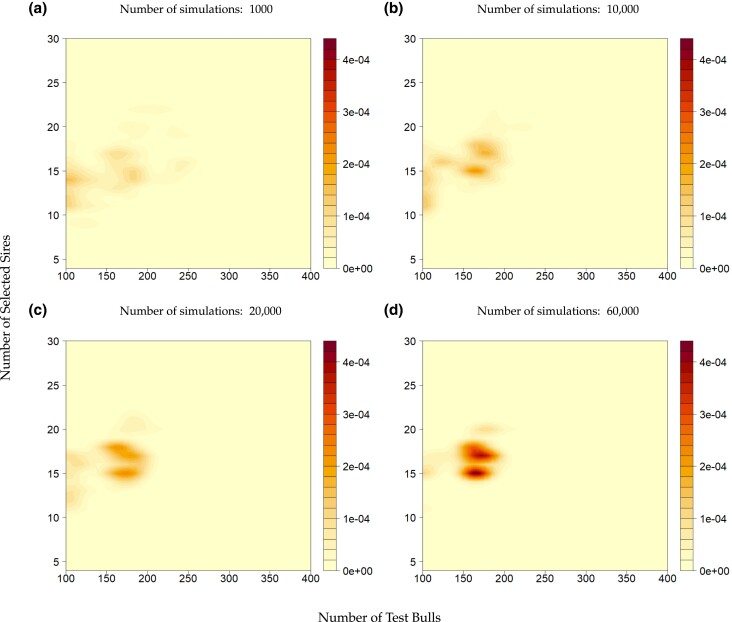
Estimates of the optimum values after smoothing using KDE associated with the number of simulations needed: a) using 1,000 simulations, b) using 10,000 simulations, c) using 20,000 simulations, d) using 60,000 simulations. Regions with darker red shades on the plot represent areas where the estimated optima are more likely to occur or are more densely distributed, whereas lighter shades suggest regions where the estimated optima are less likely to occur or are sparsely distributed.

Identifying the global maxima and, or the target area for further iterations based on the simulation of the first iteration has a significant amount of variance, due to the inherent randomness of the stochastic simulation process. When just conducting 1,000 simulations, the estimated global maximum for *m* is estimated to be in a corner solution in 21% of all runs with $100\leq x_{2}\leq 150$ (Fig. 4a). Only 18% of all estimated global maxima fall within the range ($130\leq x_{2}\leq 210$ and $15\leq x_{3}\leq 21$) that we chose for final investigation. In comparison 10,000 (Fig. 4b), 20,000 (Fig. 4c) and 60,000 (Fig. 4d) simulations result in 33%, 41%, 56% of the runs in our afterward chosen search space.

### Optimization via approximation techniques

All reported optima were computed by brute-force calculation of all potential values of the target function. However, for large-scale optimization problems with numerous parameters, this approach becomes impractical. To address this, we employed various mathematical optimization techniques on both the plain target function obtained from linear interpolation and our suggested kernel regression. When kernel regression was applied to the target function (Supplementary Table S2), all algorithms demonstrated the ability to converge to solutions that were only marginally worse than those obtained through the brute-force method.

Regarding the distance of the suggested optima to the finally obtained optima of (2377, 163, 18) in the first iteration, the GA and DEoptim algorithms resulted in a lower average distance. The optimization results obtained using the optimr algorithm in our small example demonstrated its sensitivity to the choice of the starting point. With a good initial guess ($x_{2}=200$, $x_{3}=20$), the algorithm performed satisfactorily, converging to a global optimum. However, when a slightly worse initial guess was provided, the algorithm failed to produce meaningful results (e.g. with an initial guess of ($x_{2}=250$, $x_{3}=25$), the optima found were ($x_{2}=245$, $x_{3}=23$) with a target function value of 106.986). The optimr, GA, and DEoptim algorithms exhibited superior speed, being approximately 38.85, 6.88, and 3.82 times faster than the brute-force method, respectively. The GenSA model, similar to the optimr algorithm, requires an initial guess to initiate the optimization process. Although it has the capability to converge to a good solution even with a suboptimal initial guess, it tends to take a longer time, needing approximately 5.75 times more time than the brute-force approach to reach convergence.

Similar trends were observed when the target function was interpolated without kernel regression in terms of speed (Supplementary Table S3). However, all algorithms achieved a lower value for the target function and failed to converge to a global optimum. The optimr model, with a good initial guess ($x_{2}=200$, $x_{3}=20$), performed exceptionally well in this scenario. It achieved the best solution regarding the target function and distance to the optimal solutions. On the other hand, the brute-force model performed the worst, and despite exhaustively searching through the entire solution space, it was not able to find a solution that matched the performance of the other algorithms.

## Discussion

In this study, we have developed a general optimization pipeline for breeding programs that goes beyond the limitations of traditional methods and can cope with the variability in the outcome of stochastic simulations. Insights from the results highlight five key points for discussion:

### Kernel estimator vs other optimization algorithms

The algorithm discussed in this study offers additional benefits over traditional methods like grid search when the optimal parameter region has a complex shape and the target function value has an unknown functional form but can be evaluated at any point. E.g. imagine that the optimum for a parameter is 150. If we use a grid-search algorithm that only considers a limited set of discrete values, such as 100, 200, 300, and 400, we miss the true optimum and settle for a suboptimal value of 100 or 200 as the best solution, which could lead to significant performance degradation compared to the optimal setting of 150 (Fu *et al*. 2017).

By employing kernel regression and initializing a larger random search space, a more comprehensive exploration of the problem was achieved. This approach enabled a thorough investigation of the underlying relationships and dependencies between different parameterizations, leading to a deeper understanding of the optimization landscape. This can be valuable for identifying trends or patterns that may not be immediately visible with a smaller search space.

The application of kernel regression helped overcome the challenges posed by the stochastic nature of evaluating the target function in a given parametrization. Kernel regression led to improved optimization results by the better average optimization of the target function and smaller average distances to the optimum values in Supplementary Table S2 compared to Supplementary Table S3, where the target function was interpolated. Differential evolution (Deoptim) and genetic algorithm (GA) showcased the most effective algorithms. These algorithms use principles of natural selection and genetics to explore the solution space through populations of candidate solutions and genetic operations like crossover and mutation (McCall 2005). They are known for their ability to explore a wide range of solutions and adapt to changing environments, which could be advantageous in scenarios where kernel regression alone may not be sufficient (brute-force evaluation of all parameterizations is impossible).

Similarly, the simulated annealing approach, such as GenSA, takes a more exploratory approach to optimization. It explores the search space by iteratively accepting probabilistic uphill moves, which enables it to break free from local optima and find the global optimal solution, or at least a very close approximation to it. However, this feature of accepting uphill moves comes at a cost. Simulated annealing requires a large number of iterations to explore the search space adequately. Discovering potential solutions can be computationally expensive and slow for large-scale optimization problems with many parameters (Wang and Shi 2013).

The gradient descent algorithm (optimr) relies heavily on the gradient information, making it prone to converging to nearby local optima. Such a requirement for precise starting points can make this approach highly sensitive to initial conditions, and even a slight deviation can result in suboptimal solutions (Dattner 2015). Moreover, running the local optimization algorithm multiple times from different initial conditions can become impractical, especially for complex and high-dimensional optimization problems.

Note here that one of the key downsides of kernel regression is that with the increasing number of parameters, the number of simulations needed to have good coverage of the entire search area will increase exponentially (Lavergne and Patilea 2008; Geenens 2011). Thus, optimization based on kernel regression will be limited to a relatively small number of parameters. It is crucial to weigh the benefits of adding additional parameters against the potential limitations they can impose on the accuracy and generalization of the model. Adding too many parameters can lead to overfitting and reduced model performance, as it increases both the computational complexity of the algorithm and the resources required to run it.

To address this challenge, one possible suggestion is to combine kernel regression with other powerful sequential optimization strategies using stochastic simulations, such as Bayesian optimization (Shahriari *et al*. 2016) or evolutionary algorithms (Bäck *et al*. 2000). These algorithms are known for their iterative nature, generating new solutions in each iteration, while kernel regression contributes its localized estimation capabilities. By combining the solutions from previous iterations with kernel regression, iterative optimization algorithms can guide the exploration of the search space in a more focused and efficient manner.

### The impact of bandwidth

As shown in this manuscript, kernel regression can be a powerful tool for smoothing/approximating a function with realizations impacted by stochasticity. However, it can be sensitive to the choice of bandwidth (Hardle and Marron 1985; Chen and Zitikis 2015). It is important to ensure that the bandwidth facilitates the use of appropriate counts of observations at different stages of the estimation process, as there is a well-known bias-variance trade-off for selecting the bandwidth in high or small-density areas of search space. This can have a significant impact on the accuracy and reliability of the smoothing process, as it determines the shape and width of the smoothing window. A wider *h* will result in a smoother curve with less detail, risk of systematical bias, and over-smoothing. By contrast, a narrower *h* will produce a more detailed curve with more variability.

Thus, an appropriately chosen bandwidth will weigh between those two factors. In our particular case with three variables, fitting the bandwidth via visual inspection was sufficient, facilitating our understanding of the bandwidth behavior. Based on the results, as the inbreeding level changed substantially for a few selected sires, kernel regression with large bandwidth can lead to substantial biases in the approximation. To negate this, the initial bandwidth for the genetic gain *g* was three times larger than for the inbreeding *f*. However, with many parameters, relying solely on visual inspection to determine the appropriate bandwidth choice for kernel regression is no longer applicable.

For this, various automated methods for bandwidth selection, such as cross-validation (Hardle and Marron 1985; Jones *et al*. 1996) and variance-based approaches (Brockmann *et al*. 1993), can minimize mean-squared errors and aid in selection. When using such an automated procedure for our example (particularly for the first iteration), the suggested bandwidth was smaller than what we used in this study. In general, we would recommend using conservative choices with a relatively large bandwidth in the first iteration, as the focus in the early iterations is not unbiasedness but the identification of target areas for further testing.

### Target function

Formulating an adequate target function is an important part of any decision-making breeding process (Simianer *et al*. 2021). This requires careful consideration of both short- and long-term objectives, as well as weighing the risks associated with each option, which plays an important role in determining the optimal solution to the optimization problem. In the example provided, the weighting between *g* and *f* was chosen arbitrarily, but in practice, this process requires more thoughtful consideration due to the limited options available. For example, a company focused on economic production may prioritize traits such as yield over *f* and may choose a lower weighting factor for *f* compared to a conservation breeding program, where the goal is to preserve genetic diversity, and thus the weighting factor for *f* can be higher. Besides, one could imagine a target function that reflects the economic impact of the breeding program (Nielsen *et al*. 2014), with further potential issues to quantify how much impact an improvement of e.g. fertility trait (in genomic SD units) would lead to how much increase in average annual net profit. This enables breeders to ensure that whatever resources are being spent result in expected outcomes without sacrificing too much money upfront.

Apart from calculating overall economic gains, there is considerable interest in how changes in inbreeding affect the distribution of total gains over many years. In practice, it is common to set a threshold for the maximum amount of inbreeding gain per year to ensure that the population’s genetic diversity is not compromised (Weigel and Lin 2000). In this situation, one could also consider using a target function like our exemplary case by considering genetic gain and inbreeding. This can be done by testing different penalty values placed on two objectives to find the optimal target function that results in inbreeding rates similar to those chosen for the breeding scheme or by considering a nonlinear weighting of parameters (e.g. applying a high penalty on the target function when a threshold value for a yearly inbreeding rate is exceeded). Finally, these solutions can be analyzed in detail, allowing the breeders to adjust the trade-offs between a short-term (operative) and a long-term (strategic) perspective.

Optimization problems can vary in complexity, but the number of parameters often plays a more significant role in determining the complexity of a problem, rather than the complexity of the target function itself. As the number of parameters increases, the search space grows exponentially, making it more challenging to find the optimal solution efficiently (Härdle and Müller 1997). Our attention in this study has been focused on an approach that eliminates the stochasticity aspect of the objective problem before optimization, which requires the decision-maker to weigh her or his objectives. In other words, rather than planning breeding experiments to obtain one generalized scheme, the focus is on using the kernel regression model in combination with an objective function to explore relevant conditions for a particular breeding goal and to capture complex relationships between variables.

### Number of simulations needed

The number of simulations required can be estimated from the results in Fig. 4, a–d. As a general pattern, an increased number of simulations increases the likelihood of finding the optimal or near-optimal solution (Xu *et al*. 2015). Additionally, sampling error and variance can be reduced by expending additional simulation effort to achieve a predetermined level of statistical power for the optimization strategy. However, using an unnecessarily large number of simulations wastes resources. In contrast, a small number of simulations may produce unreliable results. For basic optimization procedures, choosing fewer simulations might be feasible to obtain the desired statistical power, but making such decisions for complex optimization problems with various inputs is not straightforward, and it will be highly dependent on the breeding program at hand.

Careful consideration should be given to external factors such as time, hardware capabilities, or software availability, which cannot be directly altered by users but must still be considered when designing simulation studies. One approach that can be used successfully in this situation is to consider how much time and resources one is willing to allocate to the optimization process (L’Ecuyer and Yin 1998; Chen *et al*. 2000). As a rough guideline, we would be recommended to use at most 1/3 of the total available computing time in the first iteration. This initial iteration can be beneficial to quickly identify areas of the search space that are likely of low interest and can be disregarded for further investigations. After that, the number of simulations in subsequent iterations can be increased. This approach allows for quick progress while ensuring accuracy by focusing on areas of interest, avoiding running too many simulations in areas where the optima are not likely to be found.

Another strategy for optimization is to run a small number of simulations first and then apply kernel regression. If the results obtained from this initial analysis are not sufficiently smooth, further simulations can be performed as needed to refine the optimization process. In our example, increasing the number of simulations from 10,000 to 60,000 led to an improvement in detecting the absolute optima. However, when aiming to narrow down the search space for a second iteration, the results from both 10,000 and 60,000 simulations were very similar.

### Simulating breeding schemes: the fine line between realism and efficiency

Simulation plays a vital role in understanding the complexities of breeding schemes, but creating an accurate and efficient simulation can be challenging. A realistic simulation must consider various factors such as genetics, environmental and management conditions, and other relevant considerations. However, including too much detail can make the simulation slow and difficult to run. It is crucial to consider the purpose of the simulation and the level of detail necessary to achieve that purpose. Not all factors may be equally important, and some details that similarly affect all parametrizations might be worth to be excluded to ensure fast simulation. By making strategic choices about what details to include and what to exclude, the simulation can provide valuable insights while still being efficient to run, where the goal of the simulation should still provide an accurate representation of reality for the intended breeding purpose.

## Conclusion

In conclusion, kernel regression has proven to be a valuable tool in optimizing breeding programs with few parameters and particularly helps in coping with the stochastic nature of the target function. Its flexibility in considering a large space allows for accurate predictions, and its ability to reduce variability and refine the objective function in optimization strategies using stochastic simulations provides a more reliable assessment of potential solutions. Our finding highlights the limitations of the current optimization methods when applied to optimization problems using stochastic simulation and highlights the importance of including kernel regression. This promising strategy opens up avenues for further research in optimizing test resources and tackling larger-scale breeding optimization tasks as an intermediate step in the optimization process to maintain high accuracy while also considering practical limitations such as limited hardware capacities.

## Data Availability

Supplementary files, along with the exact model for the simulation script (Supplementary File S3), visualization script (Supplementary File S4), model comparison (Supplementary File S5), and generated data for reproducing the results have been shared with the scientific community at Figshare: https://doi.org/10.6084/m9.figshare.21996311.v1.
